# Cicero and *Burkholderia cepacia*: What’s in a Name?

**DOI:** 10.3201/eid0904.020700

**Published:** 2003-04

**Authors:** John E. Moore, Frederick Williams

**Affiliations:** * Belfast City Hospital, Belfast, Northern Ireland; † The Queen’s University of Belfast, Belfast, Northern Ireland

## Abstract

Then said they unto him, Say now Shibboleth: and he said Sibboleth: for he could not frame to pronounce it right. Then they took him and slew him at the passes of Jordan: and there fell at that time of the Ephraimites forty and two thousand.**”** Judges 12:6

In Old Testament times, mispronunciation bore a price. The Israelites (circa 1143 B.C.) used pronunciation to differentiate their own from the Ephraimites, and the consequences of mispronunciation were severe. Today, mispronunciation, though not a matter of life and death, presents problems when it interferes with communication. In scientific nomenclature, Greek or Latin bionomials of infectious disease microorganisms are often mispronounced, sometimes causing confusion among healthcare professionals (e.g., infectious disease physicians, epidemiologists, and even microbiologists). Unlike horticulturalists, who have masterfully developed a large repertoire of common names for botanical species thereby avoiding the need for and potential mispronunciation of classical Greek and Latin, infectious disease specialists still rely on Greek and Latin bionomials.

How important is a standard pronunciation of bionomials? Language is about communication. Provided the parties in a discussion can understand each other, variations in pronunciation of individual words can be tolerated or disregarded. Everyday modern English is filled with examples of variant pronunciations that cause no communication problems (e.g., either, tomato, laboratory, fertile). These variant pronunciations have many causes. Regional practice is probably the single most important variant, but educational and social backgrounds also play a part, as do personal preferences and even etymologic theories. It would be futile and (some believe) undesirable to impose uniformity by prescribing approved pronunciations when communication is not compromised. Moreover, in all languages, pronunciation changes constantly.

*Burkholderia cepacia*, an important gram-negative bacterial pathogen in patients with cystic fibrosis, may cause premature death in these patients. Since its first description in 1950 by Walter Burkholder (*1*), the pathogen has undergone several taxonomic reclassifications (*2*) in accordance with the Bacteriologic Code (1990 revision). However, uncertainty still surrounds the clinical relevance of its evolving taxonomy, particularly in regards to the nine described genomovars of the *B. cepacia* complex (BCC). The species name *cepa'cia* comes from L. fem. N. *caepa* or *cepa* (onion). Most confusion surrounding this species name was initially due to its transfer from the genus *Pseudomonas* to the newly described genus *Burkholderia* by Yabuuchi et al. in 1992 (*3*). The practice of renaming individual BBC genomovars with species names when phenotypic differentiation becomes available has heightened the confusion, as in the renaming of *B. cepacia* genomovar II to *B. multivorans,* where problems arise both with physicians (in infection control) and with patients (psychological acceptance of the disease).

Even though in *B. cepacia*, taxonomic issues rather than pronunciation are at the root of confusion, the pathogen neatly encapsulates several aspects of the linguistic conundrum involving “correct” pronunciation of Latin binomials. The correct pronunciation of both the genus *Burkholderia* and the species *cepacia* is still debated. The debate is mainly about the correct pronunciation of *cepacia*, but the genus name, *Burkholderia*, also deserves some consideration. The genus name is formed from the surname Burkholder, on which a Latin suffix *–ia* has been grafted. How should this synthetic word be pronounced? With the original pronunciation of the name retained as far as possible (long *o* and stress on second syllable) or with a more Latinized effect (short *o* and stress on third syllable, possibly lengthening its vowel)? Or is this in fact a nonexistent problem because the word is normally encountered in print so variations in pronunciation present no confusion?

The scientific and infectious disease communities would benefit from the adoption of a standard pronunciation of Latin binomials that would obviate confusion and ambiguities. The Linnaean binomial system uses Latin morphology and grammar in forming its names, and they are equally respected in China and Peru. Why not adopt a standard pronunciation? An immediate practical objection is that there is hardly a “standard Latin pronunciation.” Throughout history, Latin pronunciation has developed in accordance to the vernacular language of its users. Even as long ago as the 16th century, when Latin was of necessity the common language of such multinational organizations as the Roman Empire and the Catholic Church, speakers of Latin from different nations could not understand each other. This linguistic situation was satirized in 1528 by Erasmus, who proposed to standardize a reconstructed version of classical Latin pronunciation, i.e., the practice (as far as it could be deduced) of 1,500 years earlier. His efforts had only limited success. Two and a half centuries later, Samuel Johnson, in his Life of Milton, condemned those who, like Milton, sought to replace the “English” pronunciation with the “Italian.” A remnant of Johnson’s “English” system still persists in the Latin-derived jargon used by British lawyers. Toward the end of the 19th century, schoolmasters and classics scholars began adopting a restored pronunciation (reconstructed from heterogeneous evidence) that aimed to reproduce Latin pronunciation in the time of Cicero or Virgil (i.e., the first centuries B.C. and A.D.). This reform was supported in 1923 by a committee appointed by the Prime Minister of the United Kingdom, but the “new way” was not universally accepted.

Analogous situations are found in other European countries. In Italy, the church pronunciation still carries much prestige. In France, the reform movement encountered bitter opposition. However, the views of responsible classics scholars today seem to converge in both theoretical and practical terms. The most promising system is described by W. Sidney Allen (*4*) in which he uses symbols of the International Phonetic Alphabet. According to this system, both *c*s in *cepacia* would be pronounced hard (like English *k*); the first vowel, *e*, would be long (approximately as in Received Pronunciation of *gate*); the second, *a*, would also be long (approximately as in RP *father*); *i* (as in *dip*) and *a* would be short; and the stress would fall on the second syllable. There is some degree of artificiality in this system, since *cepacia* is not a classical word but a later scientific coinage, formed from the classical Latin *caepa*. Indeed, this scholarly pronunciation does not correspond with any current pronunciations in the scientific and infectious disease communities. Any attempt to introduce it as a standard might paradoxically cause further confusion.

The standard pronunciation of Latin that scholars have reconstructed implies the primacy (for literary purposes) of the so-called Golden Age of Caesar, Cicero, and the Augustan poets and historians. Infectious disease specialists in the 21st century should not adopt this pronunciation, unless it is a genuinely useful and acceptable solution to a real problem. Our times, unlike the era of the Israelites, do not deem mispronunciation a capital offence. Classicists should be willing to help if they are asked but have no proprietary rights over the functional idiolect of modern scientific Latin whose users can use whatever pronunciation they find conducive to communication.
